# Inactivation and Unfolding of the Hyperthermophilic Inorganic Pyrophosphatase from *Thermus thermophilus* by Sodium Dodecyl Sulfate

**DOI:** 10.3390/ijms10062849

**Published:** 2009-06-23

**Authors:** Hang Mu, Sheng-Mei Zhou, Yong Xia, Hechang Zou, Fanguo Meng, Yong-Bin Yan

**Affiliations:** 1 State Key Laboratory of Biomembrane and Membrane Biotechnology, Department of Biological Sciences and Biotechnology, Tsinghua University, Beijing 100084, China; 2 Yangtze Delta Region Institute of Tsinghua University, Jiaxing 314006, China; 3 College of Biology and Chemical Engineering, Jiaxing University, Jiaxing 314001, China

**Keywords:** hyperthermophilic enzyme, inactivation, inorganic pyrophosphatase, mixed type reversible inhibition, protein folding, sodium dodecyl sulfate, Thermus thermophilus

## Abstract

Inorganic pyrophosphatase (PPase, EC 3.6.1.1) is an essential constitutive enzyme for energy metabolism and clearance of excess pyrophosphate. In this research, we investigated the sodium dodecyl sulfate (SDS)-induced inactivation and unfolding of PPase from *Thermus thermophilus* (T-PPase), a hyperthermophilic enzyme. The results indicated that like many other mesophilic enzymes, T-PPase could be fully inactivated at a low SDS concentration of 2 mM. Using an enzyme activity assay, SDS was shown to act as a mixed type reversible inhibitor, suggesting T-PPase contained specific SDS binding sites. At high SDS concentrations, T-PPase was denatured via a two-state process without the accumulation of any intermediate, as revealed by far-UV CD and intrinsic fluorescence. A comparison of the inactivation and unfolding data suggested that the inhibition might be caused by the specific binding of the SDS molecules to the enzyme, while the unfolding might be caused by the cooperative non-specific binding of SDS to T-PPase. The possible molecular mechanisms underlying the mixed type inhibition by SDS was proposed to be caused by the local conformational changes or altered charge distributions.

## 1. Introduction

**Figure 1.**
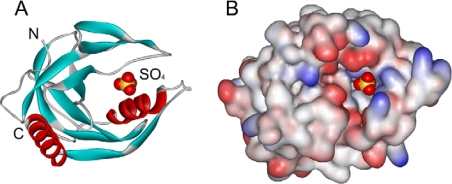
Subunit structure of PPase from *Thermus thermophilus* (T-PPase, PDB ID 2PRD). (A) Subunit structure of T-PPase by ribbon representation. The center of the active site is indicated by the position of the sulfate molecule. N and C are the N- and C-terminus of the polypeptide. (B) The cavity of the active site. The hydrophobic side chains are in white, while red and blue represent the acidic and basic side chains. The cavity is surrounded by charged residues, while the bottom of the cavity is hydrophobic. The plots were generated using WebLab ViewerLite 3.7 from Molecular Simulations.

Inorganic pyrophosphatase (PPase, EC 3.6.1.1), which specifically catalyzes the hydrolysis of the phosphoanhydride bond in inorganic pyrophosphate yielding two inorganic phosphates as the products, is an essential constitutive enzyme for energy metabolism and clearance of excess pyrophosphate [1]. It has been well established that PPase plays an important role in the regulation of the biosynthetic reactions of macromolecules including protein, RNA and DNA synthesis [1–3]. Due to its indispensable role in cellular energy metabolism, PPase is ubiquitous in nature and has been characterized from many prokaryotic and eukaryotic sources, including thermophiles. Structural analysis has revealed that PPases share a similar core structure and conserved active site though their oligomeric states differ from dimer to hexamer [4–6]. The active site of PPases is a large cavity with a diameter of around 10 Å formed by conserved polar residues, which are responsible for the binding of at least three Mg^2+^ ions and the substrate pyrophosphate (Figure 1). The bottom of the active site cavity is hydrophobic and contains a cluster of aromatic residues. The homohexameric structure of PPase from *Thermus thermophilus* HB8, which is typical of prokaryotic PPases, is packed by two trimers rotated by 30° with tight interactions between the subunits in the trimers [4].

In addition to its physiological roles as an important housekeeping enzyme, PPase also provides the simplest model reaction for phosphoanhydride bond formation and breakdown, which will help our understanding of the mechanisms of intracellular pyrophosphate and ATP turnover. PPases isolated from extremophiles has been used as a model for studies of protein thermostability [4,5]. Although the catalytic mechanism, structure and stability of PPases have been extensively studied for many years, the regulation and folding pathways of PPases have received little attention.

Sodium dodecyl sulfate (SDS) is an anionic surfactant that is frequently used in denaturing proteins, such as in the preparation of samples in SDS-PAGE [7]. For a fully-denatured protein, SDS can cooperatively bind to the protein at a high molecule ratio [8]. SDS can also specifically or non-cooperatively bind to some proteins [9–14] and in some cases, activate enzymes [15,16]. Recently, it was found that proteins behaved quite differently when denatured by moderate concentrations of SDS [17]. Moreover, it is unclear whether the hyperthermophilic proteins, which are usually considerably stable against various stresses [18], exhibit extraordinary stability against SDS-induced inactivation and denaturation. In this research, we studied the SDS-induced inactivation of PPase from *Thermus thermophilus* (T-PPase). The results indicated that although T-PPase is a hyperthermophilic enzyme, it could be fully inactivated by the existence of 2 mM SDS via a mixed type inhibition mechanism.

## 2. Results and Discussion

### 2.1. Inactivation of T-PPase by SDS

Time course experiments indicated that T-PPase inactivation by 0–2 mM SDS was completed in 2 h (data not shown). Thus the extent of T-PPase inactivation was measured by incubating the enzyme solutions with various concentrations of SDS for 2 h at 25 ºC, and then the residual activity was measured. No significant difference was observed between the data recorded using activity assay with or without the addition of the corresponding concentrations of SDS in the reaction solutions. The concentration-dependent inactivation of T-PPase by SDS exhibited a typical two-state process, and the enzyme was fully inactivated by 2 mM SDS. The midpoint of T-PPase inactivation (IC_50_) was at 1.11 ± 0.01 mM SDS.

**Figure 3.**
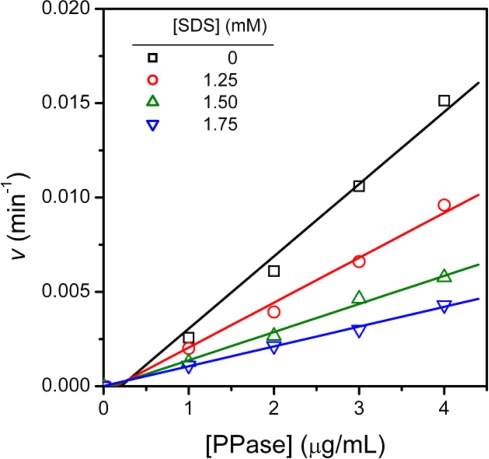
Dependence of T-PPase inactivation by SDS on enzyme concentration.

To further investigate the kinetic mechanism of SDS-induced inhibition of T-PPase activity, T-PPase inactivation by SDS was examined by varying the enzyme concentrations from 0 to 4 μg/mL in the presence of 0–1.75 mM SDS. As presented in Figure 3, at a given SDS concentration, the *v* value increased linearly with the increase of enzyme concentration, suggesting that the inhibition of T-PPase by SDS was reversible.

**Figure 4.**
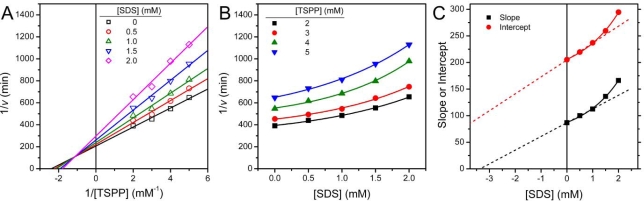
Characterization of the type of PPase reversible inhibition by SDS. (A) Lineweaver-Burk plots. (B) Dixon plots. The lines are the best non-linear regression fit of the data to the parabolic function. (C) Slopes and intercepts on the Y-axis (Y-intercept) from the double reciprocal plot were plotted as a function of SDS concentration. The solid lines are the best non-linear regression fit of the data to the parabolic function. The dashed lines are the linear fit of the three lowest SDS concentrations, which yield the apparent inhibition constants using Equations (1) and (2).

**Scheme 1.**
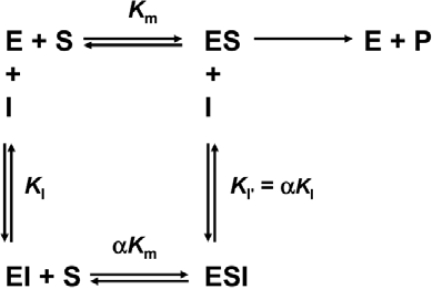
General Scheme for mixed type reversible inhibition. E, S and I denote enzyme, substrate and inhibitor respectively.

The type of T-PPase reversible inhibition by SDS was determined from the Lineweaver-Burk plots conducted in the absence or presence of different concentrations of SDS (Figure 4). In the absence of inhibitor, the enzymatic parameters of T-PPase could be derived from Figure 4A: *K*_m_ = 0.42 ± 0.04 mM and *V_max_* = 0.15 ± 0.02 min^−1^. The double reciprocal plots remained linear with the increase of [SDS], and intersected with the negative of the vertical axis (1/*v* axis) and positive of the horizontal axis. Thus the Lineweaver-Burk plot shown in Figure 4A clearly indicated that both the apparent *K*_m_ and *V_max_* values of T-PPase were altered by the existence of the inhibitor SDS. That is, the apparent *K*_m_ value increased, while the apparent *V_max_* value decreased with the increase of SDS concentrations. These observations implied that SDS was a mixed type inhibitor of T-PPase, and the mechanism is presented in Scheme 1 [19,20]. In such an inhibition mechanism, SDS displayed binding affinity for both the free enzyme and the enzyme-substrate binary complex at a site other than the active site.

The Dixon plots (plots of 1/*v* against inhibitor concentration [I]) shown in Figure 4B revealed a nonlinear relationship between 1/*v* and [SDS] and curved upward parabolically. This parabolic curve indicated that the inhibitor SDS binds to more than one site of the enzyme T-PPase. The inhibition constants *K*_I_ and *K*_I’_ were estimated from the secondary plots of the slope and vertical intercept obtained from Figure 4A against SDS concentration. Similar to the Dixon plots, a slight curvature of the replots was observed in Figure 4C, which could also be best fitted to a parabolic function. The apparent inhibition constants were estimated from the lowest three SDS concentrations, which could be regarded as an approximately linear relationship or the linear region of the replots. Then the apparent inhibition constant could be calculated from Equations. (1) and (2), which resulted in *K*_I_ = 3.4 mM and *K*_I’_ = 6.4 mM. In summary, the data in Figures 3 and 4 revealed that the inhibitory behavior of SDS was a mixed type reversible inhibition, in which the SDS molecule binds more tightly to the free enzyme than to the enzyme-substrate complex. Consequently, SDS was observed to apparently decrease the specific velocity *V*_max_ and the affinity for substrate (i.e., it increased the *K*_m_). The results also suggested that the binding of the substrate could protect the enzyme against the inhibition by SDS.

### 2.2. Structural Changes of PPase Induced by SDS

**Figure 5.**
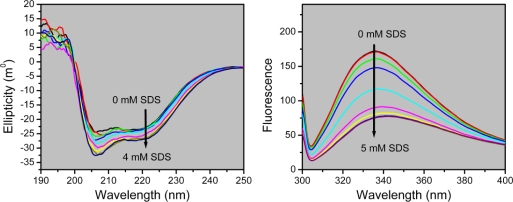
Secondary and tertiary structural changes of T-PPase during SDS-induced denaturation. The protein was incubated in buffers with the addition of various concentrations of SDS for 2 h, and then the CD or intrinsic fluorescence spectra were measured. (A) CD spectra of T-PPase denatured by various concentrations of SDS. The final enzyme concentration was 0.2 mg/mL. The arrow indicates the CD spectra are recorded in the presence of 0, 0.25, 0.75, 1.25, 1.75, 2.0, 2.5, 3.0 and 4.0 mM SDS, respectively. (B) Intrinsic fluorescence spectra of T-PPase by SDS. The excitation wavelength was 295 nm. The arrow indicates the fluorescence emission spectra measured in the presence of 0, 0.5, 1.0, 1.5, 1.75, 2.0, 2.5, 3.0, 4.0 and 5.0 mM SDS, respectively.

**Figure 6.**
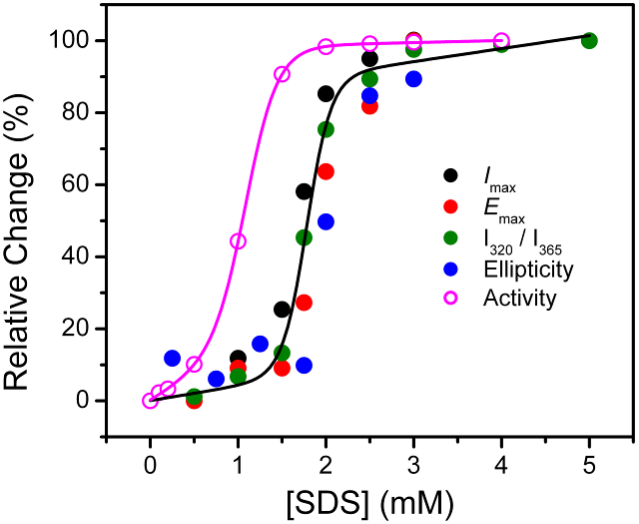
Transition curves of T-PPase unfolding by SDS monitored by the ellipticity at 222 nm, the emission maximum wavelength (*E*_max_), intensity at *E*_max_(*I*_max_) and *I*_320_/*I*_365_ of intrinsic fluorescence. The inactivation data in Figure 2 is also presented.

Far-UV CD and intrinsic fluorescence experiments were performed to investigate the secondary and tertiary structural changes during T-PPase denaturation by SDS. As presented in Figure 5A, the ellipticity slightly increased with the increasing concentrations of SDS, and the changes at 222 nm revealed a two-state transition (Figure 6). This increased helical contents by moderate concentrations of SDS has also been observed in many proteins (for example, [11,21–25]).

T-PPase contains seven Tyr residues and two Trp residues (W149 and W155), which provides a useful probe to detect the microenvironments around the aromatic residues by intrinsic fluorescence. When excited at 295 nm, the emission fluorescence was dominated by the contributions of Trp fluorophores. The fluorescence spectrum of the native T-PPase reached its emission maximum at a wavelength of 336 nm, implying that the two Trp residues were mainly exposed to bonded water [26]. With the increase of SDS concentrations, the fluorescence emission maximum wavelength (*E*_max_) red-shifted to ~343 nm, accompanied with a decrease in the intensity at *E*_max_ (*I*_max_). This observation suggested that in the presence of SDS, the Trp residues were more exposed to the solvent.

The transition curves of T-PPase unfolding by SDS were obtained by the relative changes of the ellipticity at 222 nm, *E*_max_, *I*_max_ and *I*_320_/*I*_365_, which is a sensitive tool to reflect the shape and position of fluorescence emission spectra [27], and the data are summarized in Figure 6. All probes revealed a two-state transition with similar midpoints of transition at around 1.8 mM SDS, suggesting that the SDS-induced unfolding of T-PPase was a two-state process without the appearance of any equilibrium intermediate. This opinion was also supported by the observation that the fluorescence data at 280 nm excitation coincided with those at 295 nm excitation (data not shown), which indicated that the changes of the Trp and Tyr residues were synchronous. A comparison of the inactivation and unfolding curves indicated that the loss of T-PPase activity occurred at a much lower SDS concentration than the conformational changes. This observation is quite consistent with the theory of active site flexibility proposed by Tsou [28,29].

Surfactants can bind with proteins via both hydrophobic and electrostatic interactions, and the molecular mechanism underlying protein-SDS interactions has been well-established for fully-denatured proteins [7,25]. In this case, SDS can bind to the protein at a high molecule ratio (about one SDS molecule to two amino acid residues) mainly driven by hydrophobic interactions [8]. The behavior of proteins in solutions with moderate concentrations of SDS has been proposed to involve specific binding [9–14] and to behave quite differently [17]. The results herein also suggested that SDS could specifically bind to T-PPase and act as an inhibitor. In previous reports, the inactivation of enzymes by SDS has been characterized as either irreversible [30] or noncompetitive reversible inhibition [11]. Interestingly, T-PPase inactivated by SDS was found to be a mixed type reversible mechanism with several binding sites (Figure 4). This result implied that SDS could bind with both the free enzyme and the enzyme-substrate binary complex, and the SDS binding sites were different from the active site.

The present study clearly indicated that SDS was a mixed type reversible inhibitor of T-PPase, and T-PPase inactivation occurred at an SDS concentration where the overall structure remained unchanged. Thus it seems that the inhibition effects were due to specific binding of SDS to the enzyme, which resulted in local rather than global conformational changes. The local structural changes involved the alternation of the active site, which led to a decrease in substrate binding affinity and catalytic efficiency. However, the underlying molecular mechanisms remained unclear. A close inspection of the structure of T-PPase might provide some clues. As presented in Figure 1B, the active site of T-PPase is composed of charged residues, and involves several flexible loops [4]. It is possible that the anionic SDS molecules interacted with some of the positively charged residues via electrostatic interactions. This hypothesis is quite consistent with that in a recent report, which indicates that electrostatic binding is the primary interaction for protein-SDS complex at low SDS concentrations [13]. If this is the case, the binding of the SDS molecules to the positively charged residues around the active site might alter the positions of the residues crucial for substrate binding and/or catalysis. Another possibility is that the binding of the negatively charged SDS molecules neutralized or altered the charge distribution around the active site, which could lead to a decrease in the binding affinity of the charged substrate pyrophosphate. Considering that SDS could bind to both the free and substrate-bounded enzyme with more than one binding site, both of the modified structure and altered charge distribution induced by the specific binding of SDS might be responsible for the mixed type inhibition mechanism.

## 3. Experimental Section

### 3.1. Materials

Isopropyl-1-thio-β-d-galactopyranoside (IPTG), sodium pyrophosphate (TSPP), MgCl_2_, SDS and bovine serum albumin (BSA) were purchased from Sigma. T_4_ DNA ligase, DNA Marker DL2000, Nde I and EcoR I were from TaKaRa. Pfu polymerase was purchased from iNtRON. All the other reagents were analytical grade local products.

### 3.2. Protein Expression and Purification

The full-length *T-PPase* coding sequence was cloned from the total cDNA of *Thermus thermophilus* HB27 cells by RT-PCR using Pfu polymerase and the following oligonucleotide primers: sense-primer (5’-GTCATATGATGGCGAACCTGAAGAG-3’) and anti-primer (5’-GCGAATTCC TAGCCCTTGTAGCG-3’). The obtained gene was sequenced, and the results indicated that it was the same as that from *Thermus thermophilus* HB8. The PCR product was further ligated into the expression vector pET28a (Novagen) using standard procedures, and the sequence of the product was checked by sequencing and restriction analysis. The recombinant plasmid was then transformed into *E. coli* BL21(DE3) (Novagen). The recombinant strains were grown at 37 ºC for 12 h in LB medium containing kanamycin, and then the cultures were diluted (1:100) in the same medium and grown at 37 ºC to reach an OD_600_ of 0.6. The overexpression of the recombinant proteins was induced by 1 mM IPTG. After cultivation at 24 ºC for 15 h, the cultures were harvested by centrifugation at 6,000 g for 10 min and disrupted by sonication. The soluble fractions were treated at 80 ºC for 3 h, and then the hyperthermophilic proteins were obtained by centrifugation at 15,000 g for 30 min at 4 ºC. The final products were collected from a HiLoad 16/60 Superdex 200 Prep grade column on an AKTA purification system. The protein samples were prepared in P buffer (50 mM Tris–HCl, pH 8.0 and 1 mM MgCl_2_).

### 3.3. PPase Activity Assay

The reaction solutions contained 0.5 mL P buffer, 0.02 mL TSPP stock solutions (1.7 mM) and 0.05 mL enzyme solutions. The reaction was performed at 80 ºC for 30 min and terminated by the addition of 0.1 mL citric acid with a concentration of 0.1 M. The hydrolysis of the substrate inorganic pyrophosphate was determined according to the method developed by Heinonen and Lahti [31,32]. In brief, 1.0 mL of AAM (acetone–acid–molybdate) solution was added to 0.12 mL reaction solutions. The amount of the product inorganic phosphate was determined by measuring the absorbance of phosphomolybdate at 420 nm.

### 3.4. Determination of Inhibition Constants

The inactivation of T-PPase by SDS was examined by incubating the enzyme solutions with the addition of a given concentration of SDS for 2 h at 25 ºC, and then the activity was measured as described above. The inhibition constants were determined by measuring T-PPase activity varying the substrate concentration in the absence or presence of SDS. The type of inhibition was derived from the Lineweaver-Burk plots, and the inhibition constants were calculated from the replots of the slopes and intercepts of the vertical axis according to standard methods [19,20]. For mixed type reversible inhibition, the equations used in this research were:

(1) $$\text{Slope}=K_{\text{m}}[\text{SDS}]/(V_{\text{max}}K_{\text{I}})+K_{\text{m}}/V_{\text{max}}$$

(2) $$\text{Intercept}\;=\;[\text{SDS}]/(V_{\text{max}}K_{\text{I}\prime })+1/V_{\text{max}}$$

where *K*_I_ and *K*_I’_ are the dissociation constants of the enzyme-inhibitor (EI) and enzyme-substrate-inhibitor (ESI) complexes, respectively.

### 3.5. Spectroscopy

The intrinsic fluorescence emission spectra were measured on a Hitachi F-2500 fluorescence spectrophotometer using a 1 cm path-length cuvette with an excitation wavelength of 295 nm or 280 nm. The emission spectra were collected with a wavelength ranging from 300 nm to 400 nm. Far-UV circular dichroism (CD) spectra were recorded in the range of 190–250 nm on a Jasco 715 spectropolarimeter using a cell with a path length of 0.1 cm. The final spectra were obtained by subtracting the spectra of the control (buffer solutions without the addition of protein samples). All spectra were measured at 25 ºC.

## 4. Conclusions

T-PPase was found to be fully inactivated at a low SDS concentration of 2 mM although T-PPase is a hyperthermophilic enzyme. SDS was a mixed type reversible inhibitor of T-PPase, and the inhibition might be caused by the local conformational changes and/or altered charge distributions due to the specific binding of the SDS molecules to the enzyme. At high SDS concentrations, T-PPase was denatured via a two-state process, which might be caused by the non-specific binding of the SDS molecules to the protein.

## Figures and Tables

**Figure 2.**
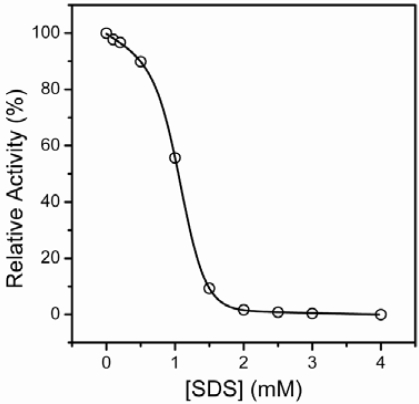
Inactivation of T-PPase by SDS. The inactivation was performed by denaturing 11.5 μg/mL enzyme by various concentrations of SDS for 2 h, and the final concentration of the enzyme was 1.0 μg/mL in the activity assay.
